# Betulinic acid increases lifespan and stress resistance *via* insulin/IGF-1 signaling pathway in *Caenorhabditis elegans*

**DOI:** 10.3389/fnut.2022.960239

**Published:** 2022-07-29

**Authors:** Haiyan Chen, Rongji Li, Feng Zhao, Li Luan, Tiantian Han, Zhong Li

**Affiliations:** ^1^College of Pharmacy, Changchun University of Chinese Medicine, Changchun, China; ^2^College of Life Sciences, Changchun Sci-Tech University, Changchun, China; ^3^College of Food Science and Engineering, Jilin Agriculture University, Changchun, China

**Keywords:** betulinic acid, *C. elegans*, lifespan, stress resistance, DAF-16

## Abstract

Numerous studies reported that betulinic acid (BA), a natural product extracted from birch bark, exhibited various beneficial effects *in vitro*. However, its pharmacological activities in aging are rarely understood. In this study, *Caenorhabditis elegans* was deployed as a whole animal model to investigate the impacts of BA on lifespan and stress resistance. Wild-type *C. elegans* were fed in the presence or absence of BA and tested for a series of phenotypes, including longevity, mobility, reproductive capacity, pharyngeal pumping, heat stress, and oxidative stress. BA at the optimal dose (50 μg/mL) extended the lifespan, improved the healthspan, and significantly evoked the increased oxidative stress resistance in *C. elegans*. Incorporating the genetic analysis with different types of longevity mutants, DAF-16, the downstream effector of the Insulin/IGF-1 receptor signaling, was revealed to mediate the protective effects of BA on lifespan and antioxidant activity. Together, these data showcased the potential of BA in promoting healthy aging, which shall facilitate its further development in the food and pharmaceutical industries.

## Introduction

Aging is one of the most significant burdens on public health. It currently accounts for about two-thirds of global deaths daily (1). In humans, aging is accompanied by complex physiological and pathological changes in almost all the organs, thus, causing shorten lifespan, reduced stress resistance, and progressive dysfunction (2). Aging research has gained milestones on the underlying mechanisms; some molecules and signaling pathways play a pivotal role in aging processes, including target of rapamycin (TOR) proteins (3), nicotinamide adenine dinucleotide (NAD+) (4), insulin-like signaling pathway (5), Chronic inflammation (6), mitochondria and oxidative stress (7). Although aging is irreversible, these discoveries have also pointed out the possible ways for aging intervention to increase lifespan and achieve healthy aging (8). For example, natural compounds with antioxidant activity, such as broccoli-derived isothiocyanate sulforaphane (9) and resveratrol in red wine (10), have been proven to exhibit anti-aging effects *via* different mechanisms.

Betulinic acid (BA), the naturally occurring lupin-type pentacyclic triterpenoid, represents another type of potential drug lead compound with antioxidant activity. BA is widely distributed in plants and most abundant in birch bark. Birch bark and its derivatives have been used in traditional medicines to treat gastrointestinal ailments such as diarrhea and dysentery in China, Russia, the United States, and other countries (11). The previous works have consistently documented the beneficial effects of BA in antioxidant, anti-inflammatory, antibacterial, antiviral, antidiabetic, antimalarial, anti-HIV, and antitumor activities (12, 13). Notably, while numerous studies have showcased the great value of BA in alleviating oxidative damages *in vitro*, the *in vivo* evidence is rare, and there is no functional assessment at the whole animal level. As a result, it is still unknown whether BA has an anti-aging effect or not.

In the past three decades, *Caenorhabditis elegans* (*C. elegans*) has become a premier animal model for aging research. As an intact multicellular organism, the short lifecycle, tractable genetics, and behavioral phenotypes decline with aging, making *C. elegans* an excellent system for investigating the environmental and genetic basis of lifespan. Some unprecedented advances in understanding the aging process stem from seminal discoveries in *C. elegans* and then translated to higher organisms, such as the regulation of longevity by DAF-2/DAF-16/insulin/insulin-like growth factor signaling (5). Moreover, *C. elegans* also has profound implications for dissecting mitochondrial longevity pathways (14), reproductive aging (15), oxidative stress (16), and dietary restriction (17). In conclusion, *C. elegans* offers substantial advantages for studying the basic biology of aging and the translational applications of anti-aging.

In this study, *C. elegans* was implemented to investigate the pharmacological activities of BA in lifespan and stress resistance. BA exhibited dose-dependent lifespan extension. The healthy aging-related behavioral phenotypes, including locomotion, pharyngeal pumping, heat stress, and oxidative stress, were also improved at the optimal dose. After testing with the longevity mutants affecting mitochondria metabolism, dietary restriction, reproductive capacity, or insulin/IGF-1 receptor signaling, DAF-16, the downstream effector of the insulin/IGF-1 receptor signaling, was discovered to mediate the protective effects of BA on longevity and antioxidant activity. The findings showcased the potential of BA in promoting healthy aging, which will facilitate its further development in the food and pharmaceutical industries.

## Materials and methods

### Chemicals and reagents

Betulinic acid (BA, 98% purity, Aladdin, Shanghai, China), total superoxide dismutase (T-SOD; R22262), catalase (CAT; R30337), malondialdehyde (MDA; R21869), and glutathione peroxidase (GSH-Px; R21876) assay kits were purchased from Shanghai Yeyuan Bioengineering Research Institute (Shanghai, China). 2′, 7′-dichlorofluorescein diacetate (DCFH-DA), and 5-hydroxy-1,4-naphthalenedione (juglone) were purchased from Sigma-Aldrich Chemical Co. (St Louis, MO, United States). All chemicals and solvents were analytical grades or higher.

### Nematode strains and maintenance

*Caenorhabditis elegans* strains were provided by Caenorhabditis Genetics Center (CGC, University of Minnesota, Minneapolis, MN, United States) and maintained using standard protocols at 20°C. The following mutant alleles and transgenes were used: wild-type Bristol N2 isolate, *eat-2 (ad1116)*, *daf-2 (e1368)*, *clk-1 (qm30)*, *glp-1 (e2141)*, *daf-16 (mgDf50)*, *skn-1 (zu67)*, *sod-1 (tm776), sod-2 (gk257), sod-3 (tm760), sod-4 (gk101), sod-5 (tm1146), ctl-1 (ok1242), ctl-2 (ok1137), ctl-3 (ok2042)*, and GR1352 (*daf-16 [mgDf47] I; xrIs87* [P_*daf–*16_GFP]).

### Toxicity assay

Betulinic acid is readily soluble in dimethyl sulfoxide (DMSO) but not in water (18). Therefore, DMSO was used to dissolve BA to make the stock solution, which was then diluted in LB liquid medium containing *Escherichia coli OP50*. It was ensured that the concentration of DMSO in the medium did not exceed 0.3% since studies have shown that DMSO content lower than 0.3% does not affect the health and lifespan of *C. elegans* (19). The synchronized *C. elegans* at the L4 stage were transferred to NGM plates containing appropriate working doses of BA (10, 50, 100, 200, 500, and 1,000 μg/mL) (20). The 0.3% DMSO group was used as vehicle control, and the pure blank control was also set. The age-synchronized animals (30 animals/well and three wells/condition replicates) were transferred to 300 μL of liquid NGM medium in 24-well plates supplemented with BA. The experiment was performed at 20°C, and the survival rate was scored every hour. The experiment was repeated three times, and each experimental group included at least 90 worms.

### Lifespan assay

According to the drug toxicity assay (Section “Toxicity assay”), the optimal dose of BA was set at 50 μg/mL. FUDR (12.5 mg/mL) was added to the medium to inhibit the egg-laying of worms. The synchronized animals were picked into the medium at the L4 stage, 60 animals/well and three wells/condition replicates (21). The day of transfer was recorded as Day 0, and the counts were scored daily. When it was observed that the animals no longer wriggled independently, a heated platinum wire hovered over the animals but without contact. Those animals that did not respond to stimuli were scored as dead. The experiment ended when all the animals died in each group.

### *Escherichia coli OP50* metabolism experiment

*Escherichia coli OP50* bacterial solution (100 mL) was inactivated by incubation at 121°C for 20 min. The inactivated bacterial solution was dispensed into sterile centrifuge tubes and centrifuged at 4,000 rpm/min for 5 min. Two-thirds of the supernatant volume was removed under aseptic conditions. The remaining bacterial solution was sonicated for 5 min, thoroughly and evenly mixed, and then added dropwise to the surface of the NGM plate (22). The synchronized animals were grown into adults and transferred to the inactivated bacteria plates of the experimental group (50 μg/mL BA) and the control group. The lifespan assay was carried out according to Section “Lifespan assay.”

### Reproductivity, mobility, pharyngeal pumping, and lipofuscin assays

For the reproductivity assay, the synchronized *C. elegans* were grown into gravid adults and then transferred to fresh medium daily in egg-laying. The total numbers of eggs were scored according to their hatching rates with or without BA application.

For the mobility assay, the synchronized *C. elegans* were grown to the L4 stage and then transferred to the appropriate medium. The day of transfer was recorded as Day 0, and the locomotion of the *C. elegans* was quantified on the 4th, 8th, and 12th days. A sinusoidal motion was defined as a one-wavelength shift relative to the long axis of the body (23). The mobility was designated to three levels as following standards*:* Class A, animals move spontaneously and smoothly; Class B, animals don’t move unless stimulated; Class C, animals don’t move but wiggle their noses or tails in response to touch (24).

For the pharyngeal pumping assay, the synchronized *C. elegans* were visualized at the magnification of 50X under a stereomicroscope. The pumping frequency of the terminal pharyngeal bulb was counted for 30s at room temperature (25).

For the lipofuscin assay, *C. elegans* were anesthetized on agarose pads with 2% sodium azide. The autofluorescence of lipofuscin *in vivo* was detected with an inverted fluorescence microscope using 355/445 nm (Ex/Em) and 550/605 nm (Ex/Em). The fluorescence intensity was analyzed using ImageJ 8.5 software.

### Stress resistance assays

Prior to stress exposure, the synchronized *C. elegans* were grown up to the L4 stage in the presence or absence of BA (50 μg/mL). FUDR (12.5 mg/mL) was added to inhibit egg-laying.

#### Thermal shock assay

*Caenorhabditis elegans* were put into incubators at 35, 38, or 40°C. Their viability was scored every hour until all the animals died (26). The experiment was repeated three times.

#### Juglone-induced oxidative stress assay

Wild-type *C. elegans* and the indicated mutants (*clk-1*, *daf-16*, *eat-2*, and *glp-1)* were put into the NGM plates containing 200 μM juglone. Their viability was scored every hour until all the animals died (27). The experiment was repeated three times.

### Antioxidant enzyme activity and reactive oxygen species determination in *Caenorhabditis elegans*

The synchronized *C. elegans* were fed in the presence or absence of BA and grew up to the L4 stage. After three times washing with M9 buffer, the animals were collected by high-speed centrifuge and lysed for protein extraction. The protein concentrations were quantified to ensure consistency, and the supernatants of each sample were added to the 96-well plate. The kits for malondialdehyde (MDA), total superoxide dismutase (T-SOD), glutathione peroxide (GSH-Px) kit, and catalase (CAT) were used to measure antioxidant enzyme activities and lipid oxidation levels following the corresponding instructions.

To detect ROS with DCFH-DA staining, the L4-stage *C. elegans* were washed and collected into the centrifuge tube. A 200 μL aliquot of 10 mM DCFH-DA solution was added, and the tube was shaken gently to make animals in contact with the dye solution evenly. The staining lasted for 30 min at 37°C and was stopped by discarding the dye solution. The animals were washed with M9 buffer and anesthetized with levamisole. The animals were loaded onto agarose pads, and the images were captured with an inverted fluorescence microscope using 504/529 nm (Ex/Em). The fluorescence intensity was analyzed using ImageJ 8.5 software.

### Effects of betulinic acid on the expression levels of related antioxidant genes in *Caenorhabditis elegans*

The synchronized *C. elegans* were fed in the presence or absence of BA and grew up to the L4 stage. After three times washing with M9 buffer, the animals were collected by high-speed centrifuge. The total RNA was extracted using the TransZol Up Plus RNA Kit and was reverse-transcribed into cDNA using the EasyScript One-Step gDNA Removal and cDNA Synthesis SuperMix Kit. The expression of antioxidant genes (*ctl-1, ctl-2, ctl-3, sod-1, sod-2, sod-3, sod-4*, and *sod-5)* was detected using the 2^–ΔΔ*Ct*^ method. The primer sequences of the RT-qPCR reaction are shown in Table 1.

**TABLE 1:** RT-qPCR primer details.

Gene	Forward	Reverse
*ctl-1*	5′-TACATTGCTCGAAGTG CCGA-3′	5′-TCAGATGGTAGCGGCG AATC-3′
*ctl-2*	5′-TGATCGAGGTCGGCA AGATG-3′	5′-AGGCGGATTGTTCAAC CTCA-3′
*ctl-3*	5′-CTCTTGCTGAGCCAAT CCGT-3′	5′-TGGCCAAATGGAGTCG TTGG-3′
*sod-1*	5′-ATTCGTCGACGCGGAA AGAA-3′	5′-CTTTAATAAGGTTTCGA CCGA-3′
*sod-2*	5′-CGAGTCTCGAAGGTCT GCTG-3′	5′-ACGGTCGGAGCAAAC TGTG-3′
*sod-3*	5′-TCTCCAACCAGCGCTG AAAT-3′	5′-GAACCGAAGTCGCGCT TAAT-3′
*sod-4*	5′-TCTCCAACCAGCGCT GAAAT-3′	5′-GAACCGAAGTCGCGCT TAAT-3′
*sod-5*	5′-GATCGTCACCAAGTC CGAGG-3′	5′-GGAATCCGGAGAGCAT CCAG-3′
*dod-17*	5′-GTTTGGTGGCCACTTC CAAC-3′	5′-AGCTCGTTTCGTTCT CAGGG-3′

### Gene knock-down experiment

Gene knock-down was achieved by RNAi strategy. The plasmid pL4440 [Tiangen Biotech (Beijing) Co., Ltd., Beijing, China] was used to construct the *E. coli* strain for the induction of RNAi by feeding in *C. elegans.* In brief, the cDNA of *daf-16* was cloned into the L4440 plasmid and then transformed into the HT115 *E. coli* strain. The RNAi-feeding bacteria were cultured and harvested. *C. elegans* were fed with the bacteria to achieve RNAi of the *daf-16* gene. The gene knock-down efficiency was confirmed by RT-qPCR of *daf-16*. To determine the lifespan of *daf-16* RNAi strain, the experimental procedures were described in Section “Reproductivity, mobility, pharyngeal pumping, and lipofuscin assays.” To analyze the downstream genes (*clk-1*, *ctl-2*, *isp-1*, *dod-17*, and *sod-3*) affected by *daf-16* RNAi, the RT-qPCR method was described in Section “Effects of betulinic acid on the expression levels of related antioxidant genes in *Caenorhabditis elegans*.”

### Statistical analysis

All the experiments were repeated at least three times. GraphPad Prism 8.0 (San Diego, CA, United States) was used for data analysis. The data of the lifespan assays and stress resistance assays were analyzed using the Kaplan-Meier method, and the *p*-values of survival differences were determined with the log-rank test. The data were presented as mean and SEM in the other groups. “*” indicates *P* < 0.05, i.e., the difference is statistically significant, “**” indicates *P* < 0.01, i.e., a significant difference, and “***” indicates *P* < 0.001, i.e., a very significant difference.

## Results

### Betulinic acid extends lifespan in *Caenorhabditis elegans*

**FIGURE 1:**
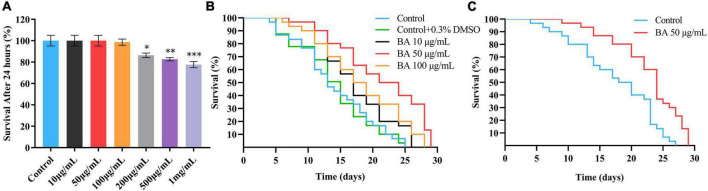
Optimal dose of betulinic acid (BA) extends lifespan of *Caenorhabditis elegans*. **(A)** Survival rates of *C. elegans* after 24 h treatment with different doses of BA (0, 10, 50, 100, 200, 500, and 1,000 μg/mL). **(B)** Lifespan analysis of *C. elegans* supplied with indicated BA (10, 50, and 100 μg/mL) or vehicle (0.3% DMSO). **(C)** Lifespan analysis of *C. elegans* fed with inactivated *Escherichia coli OP50* and BA (50 μg/mL). For panel **(A)**, comparisons represent the One-way ANOVA followed by *post-hoc* Bonferroni’s test. Data were displayed as mean ± SEM. *P < 0.05; **P < 0.01; ***P < 0.001.

**FIGURE 2:**
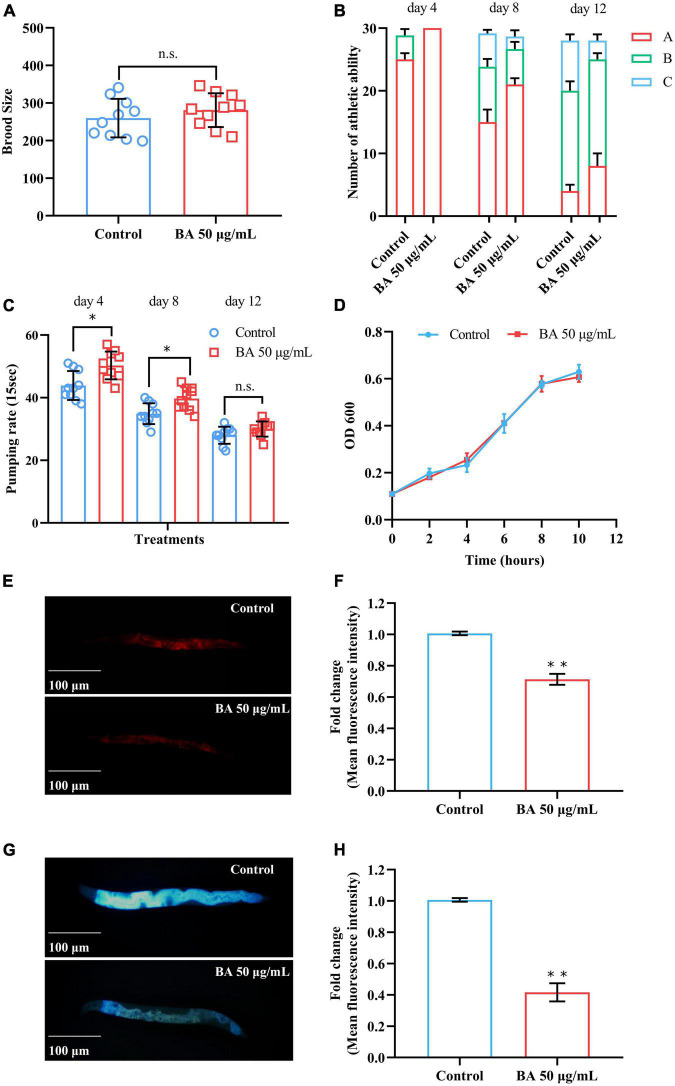
Betulinic *acid (BA) delays age-associated physiological decline.* **(A)** Reproductive capacity of *Caenorhabditis elegans* supplied. **(B)** Age-dependent motor activity of *C. elegans*. **(C)** Pharyngeal pumping rates of *C. elegans*. **(D)** Growth rate of *Escherichia* coli OP50 under 50 μg/mL BA treatment. **(E–H)** Lipofuscin induced auto-fluorescence under 550/605 nm (Ex/Em) and 355/445 nm (Ex/Em) detection. Data were displayed as mean ± SEM. For panel **(C)**, comparisons represent the One-way ANOVA followed by post-hoc Bonferroni’s test. For panels **(A,F,H)**, comparisons represent two-tailed unpaired Student’s *t*-test. *P < 0.05; **P < 0.01; ns = nonsignificant.

The study began by performing an *in vivo* assay for drug toxicity screening. The wild-type *C. elegans* were treated with various doses of BA, and their survival rates were scored 24 h later. BA did not affect the viability of animals when at the dose range of 0∼100 μg/mL (Figure 1A). Accordingly, BA at the concentrations of 10, 50, and 100 μg/mL were selected for the lifespan assay. To avoid the potential effect caused by long-term exposure to DMSO, which was used to dissolve BA, the vehicle control was set to 0.3% DMSO. As shown in Figure 2A, all three doses of BA application significantly extended *C. elegans* life span, and there was no interference from DMSO (Table 2). Notably, BA at 50 μg/mL evoked the maximal effect in extending lifespan; therefore, this optimal dose was kept for all tests down the road.

**TABLE 2:** Effects of betulinic acid (BA) on lifespan of *Caenorhabditis elegans.*

Group	Mean lifespan ± SE (days)	Change in mean lifespan (%)	Log-rank test	Median lifespan (days)
Control	13.61 ± 0.25	0		12
Control + 0.3% DMSO	13.82 ± 0.48	1.6	*P* = *0*.175	12
BA (10 μg/mL)	16.57 ± 0.37	21.79	*P* < 0.001	16
BA (50 μg/mL)	18.50 ± 0.31	35.99	*P* < 0.001	19
BA (100 μg/mL)	17.11 ± 0.42	25.72	*P* < 0.001	17

The lifespan assay requires feeding *C. elegans* with *E. coli OP50*, a living organism that may lead to unwanted metabolism of BA and non-specific phenotypes. To eliminate this possibility, the lifespan assay was carried out using the inactivated *E. coli OP50*, and the lifespan extension phenotype (Figure 1C) could be reproduced. Together, these data demonstrated that BA exhibits the ability to extend lifespan in *C. elegans*.

Summary of mean lifespan and statistical analysis for Figure 1B. Mean lifespan values were calculated by a log-rank test. *P*-values were calculated for individual experiments and each consisting of control and treatment groups.

### Betulinic acid improves healthspan in *Caenorhabditis elegans*

In general, aging affects both lifespan and healthspan, which can be measured by the functional decline in performance tests (28). The behavioral and functional phenotypes closely associated with aging were surveyed. The first was reproductive capacity, which has been considered inseparable from the lifespan (29). For anti-aging research, it is important to evaluate the brood size of *C. elegans to determine* whether BA damage the reproductive ability. As shown in Figure 2A, BA prolonged longevity without sacrificing reproductive capacity. The second phenotype, mobility, reflects the motor ability of animals and declines in the aging process. *C. elegans* motility was scored on the 4th, 8th, and 12th days according to the three-grade score: A, the animals moved spontaneously and smoothly; B, the animals did not move unless stimulated; C, the animals only wiggled their nose or tail in response to touch. Clearly, *C. elegans* motility decreased over time while BA application ameliorated this phenotype (Figure 2B).

Some diets and drugs may affect the feeding efficiency of *C. elegans*, which will lead to a dietary restriction (DR) effect that prolongs their lifespan (30). Therefore, the pharyngeal pumping rates were examined to determine the food intake behavior of *C. elegans*. Intriguingly, BA application enhanced the pharyngeal pumping rates on the 4th and 8th day. However, there was no significant difference on day 12 (Figure 2C). In light of the potential bacteriostasis of BA, which may cause non-specific dietary restriction, the growth of *E. coli OP50* was evaluated, and this scenario was also ruled out (Figure 2D). This data indicated that dietary restriction was not a primary factor in BA anti-aging effect.

Lipofuscin is an auto-fluorescent substance that accumulates during the aging process due to the elevated levels of oxidized proteins in lysosomes (31). Lipofuscin produces red fluorescence that gradually accumulates as *C. elegans* grows, whereas blue fluorescence occurs before animals die. BA application decreased the intensity of both red and blue fluorescence (Figures 2E–H), consisting of its antioxidant activity from *in vitro* studies. Together, these results demonstrated that BA promotes healthspan in *C. elegans*.

### Betulinic acid enhances heat stress resistance in *Caenorhabditis elegans*

**FIGURE 3:**
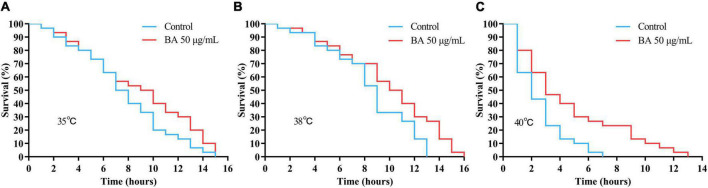
Effects of betulinic acid (BA) on heat stress resistance in *Caenorhabditis elegans.* Wild-type *C. elegans* were exposed to heat shock stress as indicated: **(A)** 35°C; **(B)** 38°C; **(C)** 40°C. The protective effects of BA were analyzed by Kaplan-Meier survival curves.

*Caenorhabditis elegans* is extremely sensitive to changes in temperature. It has been proved that higher temperature results in faster senescence. For example, *C. elegans* life cycle grown at 25°C is 2.1 times shorter than those at 16°C (26). On the other hand, the improvement in *C. elegans* ability to resist heat stress usually corresponds to an extension of their lifespan. Wild-type animals were subjected to heat stress at 35, 38, and 40°C, and their viability was scored. BA application dramatically enhanced the heat stress resistance in all three conditions and was much more pronounced at high temperatures (Figure 3 and Table 3).

**TABLE 3:** Effects of betulinic acid (BA) on heat stress resistance in *Caenorhabditis elegans.*

Temperature	Group	Mean lifespan ± SE (hours)	Change in mean lifespan (%)	Log-rank test	Median lifespan (hours)
35°C	Control	7.72 ± 0.34			7
	50 μg/mL BA	7.82 ± 0.46	1.29	*P* = 0.1321	8
38°C	Control	6.90 ± 0.47			7
	50 μg/mL BA	8.98 ± 0.31	30.14	*P* < 0.001	*9*
40°C	Control	2.73 ± 0.42			3
	50 μg/mL BA	3.85 ± 0.38	41.02	*P* < 0.001	4

Summary of mean lifespan and statistical analysis for Figure 3. Mean lifespan values were calculated by a log-rank test. *P*-values were calculated for individual experiments and each consisting of control and treatment groups.

### Betulinic acid enhances oxidative stress resistance in *Caenorhabditis elegans*

**FIGURE 4:**
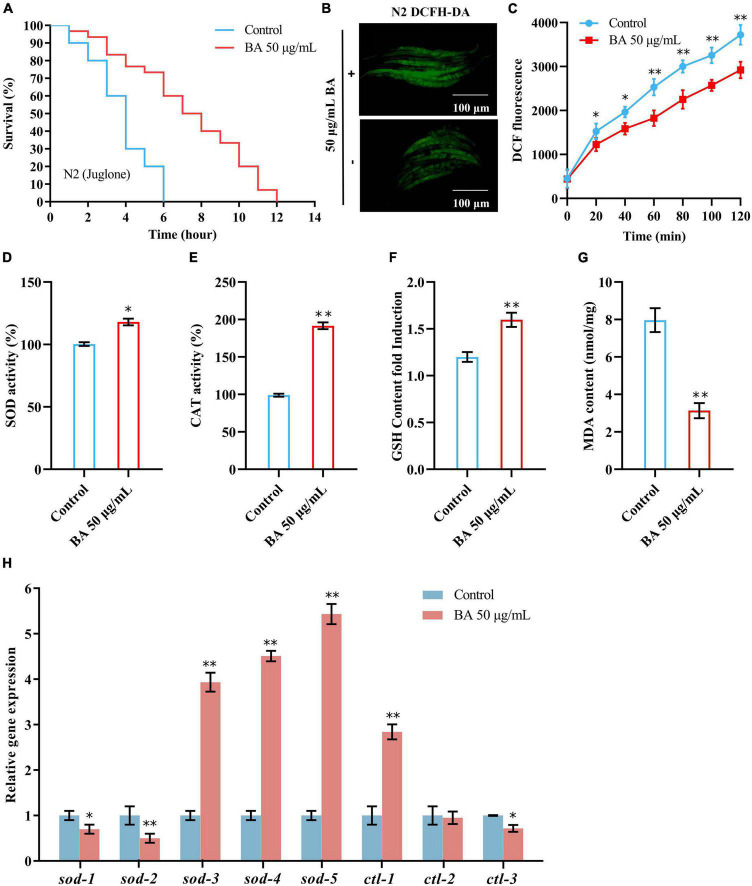
Effects of betulinic acid (BA) on oxidative stress in *Caenorhabditis elegans*. **(A)** Effects of BA on juglone-induced oxidative stress. **(B,C)** BA decreased ROS levels in *C. elegans.* Representative images of DCFH-DA staining **(B)** and quantification of fluorescence intensity **(C)** were presented. **(D–G)** Levels of lipid oxidation: SOD activity **(D)**, CAT activity **(E)**, MDA content **(F)**, and GSH-Px content **(G)**. **(H)** RT-qPCR detection of the expression of antioxidant genes. Data were displayed as the mean ± SEM. For panels **(C,H)**, comparisons represent the One-way ANOVA followed by *post-hoc* Bonferroni’s test. For panels **(D–G)**, comparisons represent two-tailed unpaired Student’s *t*-test. *P < 0.05; **P < 0.01.

The free radical theory of aging is the most consistent across experimental data and model systems. *C. elegans* advocates that cell and tissue injury by oxidative stress contributes to the aging process. As shown in Figure 4A, juglone, a commonly used oxidative stress inducer (32), led to a quick death within 6 h in *C. elegans*. However, BA application extended their survival time to 12 h (Table 4), confirming that BA has robust antioxidant activity. Moreover, this was also visualized by detecting the reactive oxygen species (ROS) in the *C. elegans* body. DCFA-DA staining showed that ROS accumulated rapidly over time, while BA exhibited a consistent inhibitory effect (Figures 4B,C).

**TABLE 4:** Effects of betulinic acid (BA) on juglone-induced oxidative stress.

Group	Mean lifespan ± SE (days)	Change in mean lifespan (%)	Log-rank test	Median lifespan (days)
Control	3.71 ± 0.19			4
50 μg/mL BA	5.52 ± 0.33	48.78	*P* < 0.001	6

Then, the impacts of BA on antioxidant enzymes were investigated. The activities of SOD, CAT, and GSH-Px were elevated, while the MDA content, a parameter reflecting the lipid oxidation level, decreased (Figures 4D–G). At the mRNA level, a generally similar tendency was observed. 5 SOD genes and three CAT genes were detected, and the expression levels of *sod-3*, *sod-4*, *sod-5*, and *ctl-1* were increased dramatically (Figure 4H).

The rest were mildly decreased (*sod-1, sod-2*, and *ctl-3*) or unchanged (*ctl-2*). Together, these data confirmed the robust effect of BA in enhancing oxidative stress resistance.

Summary of mean lifespan and statistical analysis for Figure 4A. Mean lifespan values were calculated by a log-rank test. *P*-value was calculated for control and treatment group.

### Anti-oxidant activity of betulinic acid is dependent on DAF-16/FOXO

**FIGURE 5:**
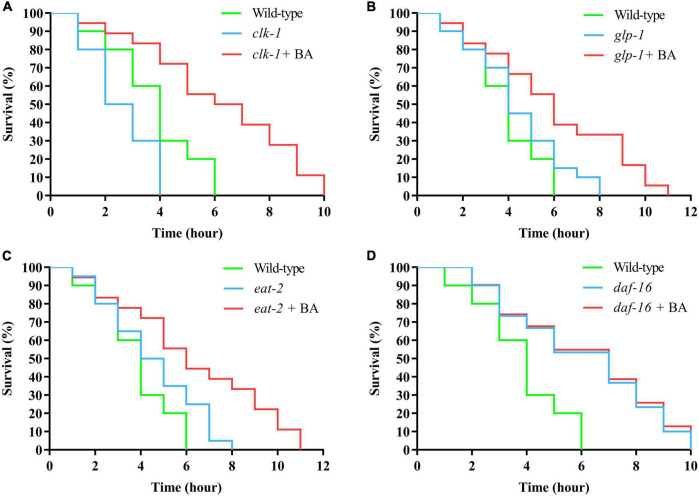
DAF-16 is required for betulinic acid (BA)-induced oxidative stress resistance. The classical longevity mutants, including *clk-1* **(A)**, *glp-1* **(B)**, *eat-2* **(C)**, and *daf-16* **(D)**, were tested for juglone-induced oxidative stress. The Kaplan-Meier survival curves were generated to analyze the effects of BA.

To dissect the underlying mechanism of BA action, the classical longevity mutants were utilized for genetic analysis: *clk-1*, the human coenzyme Q7 homolog and corresponds to the mitochondrial signaling; *glp-1*, the human notch receptor homolog and corresponds to the reproductive restriction signaling; *eat-2*, the human α7 nicotinic receptor subunit homolog and corresponds to the dietary restriction signaling; *daf-16*, the human FOXO homolog and corresponds to the insulin signaling pathways. Juglone-induced oxidative stress experiments were performed, and BA applications were added on top of the above mutants. Interestingly, BA enhanced oxidative stress resistance in the conditions of *clk-1*, *glp-1*, and *eat-2* (Figures 5A–C). However, this effect was absent when DAF-16 was loss-of-function (Figure 5D). Thus, BA depends on the insulin signaling pathway to mediate its effects against oxidative stress in *C. elegans* (Table 5)

**TABLE 5:** DAF-16 is required for betulinic acid (BA)-induced oxidative stress resistance.

Group	Mean lifespan ± SE (days)	Change in mean lifespan (%)	Log-rank test	Median lifespan (days)
Wild-type	3.71 ± 0.19			3
*clk-1*	2.62 ± 0.41	–29.38	*P* < 0.001	*2*
*clk-1* + BA	6.22 ± 0.51	67.83	*P* < 0.001	5
*glp-1*	4.42 ± 0.28	19.13	*P* < 0.001	4
*glp-1* + BA	6.01 ± 0.55	61.99	*P* < 0.001	6
*eat-2*	4.55 ± 0.26	22.64	*P* < 0.001	4
*eat-2* + BA	6.33 ± 0.31	45.98	*P* < 0.001	6
*daf-16*	6.06 ± 0.19	63.34	*P* < 0.001	5
*daf-16* + BA	6.19 ± 0.25	66.84	*P* < 0.001	5

Summary of mean lifespan and statistical analysis for Figure 5. Mean lifespan values were calculated by a log-rank test. *P*-values were calculated for individual experiments and each consisting of wild-type, mutant, and treatment groups.

### Insulin/IGF-1 receptor signaling mediates betulinic acid-induced longevity and stress resistance

**FIGURE 6:**
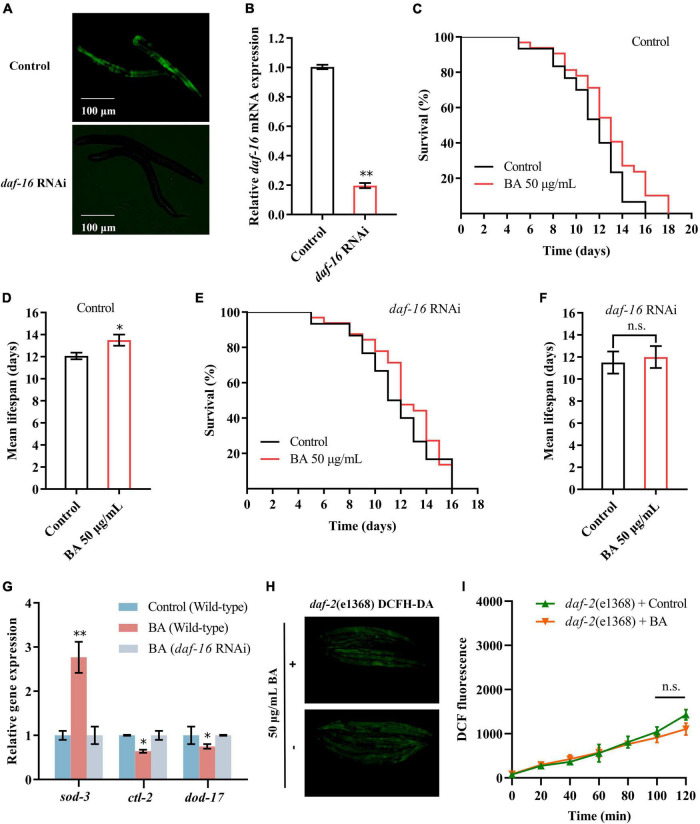
Insulin/IGF-1 receptor signaling mediates betulinic acid (BA)-induced longevity and stress resistance. **(A)** Evaluation of *daf-16* RNAi efficiency using the transgenic *Caenorhabditis elegans* expressing DAF-16:GFP. **(B)** RT-qPCR quantification of *daf-16* mRNA levels after RNAi. **(C,D)** Kaplan-Meier survival curve and mean lifespan of BA-induced longevity in wild-type animals. **(E,F)** Kaplan-Meier survival curve and mean lifespan of BA-induced longevity in *daf-16* RNAi animals. **(G)** Effects of BA on the expression of *daf-16* downstream genes (*sod-3*, *ctl-2*, and *dod-17*). **(H,I)** Effects of BA on ROS level in wild-type animals and *daf-2* mutants. Representative images of DCFH-DA staining **(H)** and quantification of fluorescence intensity **(I)** were presented. Data were displayed as the mean ± SEM. For panels **(G,I)**, comparisons represent the One-way ANOVA followed by *post-hoc* Bonferroni’s test. For panels **(B,D,F)**, comparisons represent two-tailed unpaired Student’s *t*-test. *P < 0.05; **P < 0.01; ns = nonsignificant.

The DAF-16/FOXO transcription factor is the major downstream output of the insulin/IGF-1 receptor signaling pathway. The RNAi strategy was deployed to evaluate the pivotal role of DAF-16/FOXO in BA-induced longevity and stress resistance. The knock-down efficiency was measured by quantifying the fluorescence intensity of DAF-16:GFP (Figures 6A,B). Consistent with the previous result (Figure 5D), *daf-16* RNAi eliminated the prolonged lifespan induced by BA (Figures 6C–F). The target genes of DAF-16, *sod-3, ctl-2*, and *dod-17* and their altered expression induced by BA application were abolished (Figure 6G).

To further validate the involvement of the insulin/IGF-1 receptor signaling, DAF-2, the human IGF-1 receptor homolog in *C. elegans*, was examined. As shown in Figures 6H,I, there was no difference between the control and BA application in DCFA-DA staining detected ROS levels. Together, insulin/IGF-1 receptor signaling is required for BA-induced longevity and stress resistance.

## Discussion

Betulinic acid has a wide range of physiological activities, and numerous efforts have been made to pursue its applications in food and pharmaceutical industries. First of all, the isolation and synthesis of BA have progressed significantly. For example, the current researchers extracted BA from birch bark and achieved a purity of 97.6% (33). As a lupin-type pentacyclic triterpenoid, BA is easily soluble in solvents such as DMSO, pyridine, and tetrahydrofuran but is insoluble in water. It has been known that DMSO is slightly toxic when serving as a vehicle for treating *C. elegans* (34). Therefore, the dose usage of BA dissolved in DMSO was carefully checked. It was determined that DMSO below 0.3% did not affect the health and growth of *C. elegans*; furthermore, the optimal dose of BA exhibited the maximal effect in lifespan extension.

The advantages of *C. elegans* model in aging research have been well-documented (35). As an intact animal, there are multi-dimensional aging-related readouts not only reflecting lifespan but also healthspan. The behaviors, including reproductivity, motility, pharyngeal pumping, and stress resistance, are declined over age. Hence, *C. elegans* offers a great chance to look for biological and pharmacological interventions in anti-aging. A large set of new regulators and natural compounds were initially found to prevent healthy aging in C. elegans and then further verified in higher models (36–39). Based on *C. elegans* model, a systemic evaluation was performed to investigate BA effects on healthspan. BA did not affect the reproductive ability or pharyngeal pumping rate; instead, it enhanced motor ability and heat stress resistance remarkably. These are vital signs to indicate BA has the potential to improve healthspan. This assumption was further validated by the in-depth analysis of oxidative stress and ROS.

Reactive oxygen species are highly reactive oxygen molecules that induce genotoxicity and physiological damages that destroy the innate stress resistance mechanisms. It leads to DNA damage, alters gene expression, perturbs cellular signaling, and ultimately results in cell senescence and death (40). The level of ROS in cells is a balance between ROS production by stressors and ROS by antioxidants. Therefore, reduction in ROS levels is inseparable from the regulation of antioxidant enzyme inhibitors such as T-SOD, MDA, CAT, and GSH-Px (41). Notably, BA application evoked the high contents and activities of these enzymes and then suppressed ROS accumulation in various scenarios. These results are consistent with the previous *in vivo* and *in vitro* data (42–45). In conclusion, BA exhibited a robust antioxidative effect.

So far, multiple signaling pathways are known to regulate lifespan extension (46). Therefore, the pathway responsible for aging and anti-aging can be screened in *C. elegans* efficiently (47). The insulin/IGF-1 receptor signaling pathway is a hub that integrates many upstream and downstream factors (48). In search of the molecular pathway mediating BA action, DAF-16/FOXO, the primary output of insulin/IGF-1 receptor signaling in *C. elegans*, determines BA-induced longevity and stress resistance. The pathway was further validated by the genetic analysis of upstream receptor DAF-2 and downstream targets *sod-3, ctl-2*, and *dod-17.* DAF-2, DAF-16, and the insulin/IGF-1 receptor signaling pathway are highly conserved across species. Therefore, it is reasonable to speculate that BA may have similar functions in higher organisms. In fact, BA has been implicated in several insulin/IGF-1 receptor signaling-related phenotypes. For example, BA has a potent insulin secretagogue effect in pancreatic islets (49) and exhibits the anti-diabetic effect on mouse model (50). BA improves insulin sensitivity in metabolic syndrome rats (51), but inhibits insulin/IGF-1 receptor signaling to suppress *de novo* lipogenesis in HepG2 cells (52). Although the molecular mechanisms remain to be elucidated, the current studies have provided appreciable evidence showing that insulin/IGF-1 receptor signaling plays a pivotal role in BA’s pharmacological activities.

## Strengths and limitations

The present study deployed *C. elegans* model and revealed the anti-aging effect of BA for the first time. The finding that insulin/IGF-1 receptor signaling mediates BA function will reinforce further development of BA in food and pharmaceutical applications.

Two significant limits need to be solved in the future:

1. It is unclear whether BA works through insulin/IGF-1 receptor signaling directly or indirectly.
2. Additional studies in invertebrate and mammalian model organisms are necessary to validate and expand the anti-aging effect of BA.

## Conclusion

1. Betulinic acid prolongs the lifespan and improves the healthspan in *C. elegans*.
2. Betulinic acid has robust antioxidant activity *in vivo*.
3. The insulin/IGF-1 receptor signaling pathway mediates BA-induced longevity and oxidative stress resistance.

## Data availability statement

The original contributions presented in this study are included in the article/supplementary files, further inquiries can be directed to the corresponding author.

## Author contributions

RL: conceptualization, methodology, writing—review and editing, and supervision. FZ: data curation and formal analysis. LL: validation and project administration. TH: software and supervision. ZL: writing—review and editing. HC: supervision, project administration, funding acquisition, and writing— review and editing. All authors have read and agreed to the published version of the manuscript.
